# AhR and Arnt differentially regulate NF-κB signaling and chemokine responses in human bronchial epithelial cells

**DOI:** 10.1186/s12964-014-0048-8

**Published:** 2014-07-24

**Authors:** Johan Øvrevik, Marit Låg, Valerie Lecureur, David Gilot, Dominique Lagadic-Gossmann, Magne Refsnes, Per E Schwarze, Tonje Skuland, Rune Becher, Jørn A Holme

**Affiliations:** 1 Department of Air Pollution and Noise, Division of Environmental Medicine, Norwegian Institute of Public Health, Oslo, Norway; 2 UMR Inserm 1085, Institut de Recherche en Santé, Environnement, Travail, Rennes, France; 3 EA4427-SeRAIC, IFR140, Université de Rennes 1, Faculté des Sciences Pharmaceutiques et Biologiques, Rennes, France; 4 UMR CNRS 6290, GEO Team (Gene Expression and Oncogenesis), Institut de Génétique et de Développement de Rennes, Faculté de Pharmacie, Université de Rennes I, Rennes, France

**Keywords:** Aryl hydrocarbon receptor, AhR nuclear translocator, interleukin-8, RANTES, Nuclear factor NF-κB, p65, RelB, Inflammation, Lung epithelial cells

## Abstract

**Background:**

The aryl hydrocarbon receptor (AhR) has gradually emerged as a regulator of inflammation in the lung and other tissues. AhR may interact with the p65-subunit of the nuclear factor (NF)-κB transcription factors, but reported outcomes of AhR/NF-κB-interactions are conflicting. Some studies suggest that AhR possess pro-inflammatory activities while others suggest that AhR may be anti-inflammatory. The present study explored the impact of AhR and its binding partner AhR nuclear translocator (Arnt) on p65-activation and two differentially regulated chemokines, CXCL8 (IL-8) and CCL5 (RANTES), in human bronchial epithelial cells (BEAS-2B).

**Results:**

Cells were exposed to CXCL8- and CCL5-inducing chemicals, 1-nitropyrene (1-NP) and 1-aminopyrene (1-AP) respectively, or the synthetic double-stranded RNA analogue, polyinosinic-polycytidylic acid (Poly I:C) which induced both chemokines. Only CXCL8, and not CCL5, appeared to be p65-dependent. Yet, constitutively active unligated AhR suppressed both CXCL8 and CCL5, as shown by siRNA knock-down and the AhR antagonist α-naphthoflavone. Moreover, AhR suppressed activation of p65 by TNF-α and Poly I:C as assessed by luciferase-assay and p65-phosphorylation at serine 536, without affecting basal p65-activity. In contrast, Arnt suppressed only CXCL8, but did not prevent the p65-activation directly. However, Arnt suppressed expression of the NF-κB-subunit RelB which is under transcriptional regulation by p65. Furthermore, AhR-ligands alone at high concentrations induced a moderate CXCL8-response, without affecting CCL5, but suppressed both CXCL8 and CCL5-responses by Poly I:C.

**Conclusion:**

AhR and Arnt may differentially and independently regulate chemokine-responses induced by both inhaled pollutants and pulmonary infections. Constitutively active, unligated AhR suppressed the activation of p65, while Arnt may possibly interfere with the action of activated p65. Moreover, ligand-activated AhR suppressed CXCL8 and CCL5 responses by other agents, but AhR ligands alone induced CXCL8 responses when given at sufficiently high concentrations, thus underscoring the duality of AhR in regulation of inflammation. We propose that AhR-signaling may be a weak activator of p65-signaling that suppresses p65-activity induced by strong activators of NF-κB, but that its anti-inflammatory properties also are due to interference with additional pathways.

## Introduction

The aryl hydrocarbon receptor (AhR) is a ligand-activated transcription factor originally discovered as a receptor for 2,3,7,8-tetrachlorodibenzo-p-dioxin (TCDD) and other environmental pollutants, including polycyclic aromatic hydrocarbons (PAHs). In its classical mode of action, cytosolic AhR activated by hydrocarbons rapidly translocates to the nucleus where it dimerizes with the AhR nuclear translocator (Arnt). The AhR/Arnt complex binds to dioxin or xenobiotic response elements (DREs or XREs) in the promoter region of target genes, such as the cytochrome P450 enzymes CYP1A1 and -1B1 [1].

It has now become evident that the AhR is also involved in regulation of inflammation as well as a variety of other endogenous processes. Chronic low-grade inflammation is a common feature of a variety of multi-factorial diseases including various pulmonary disorders [2],[3]. It has been suggested that increases in such chronic disorders at least partly may be related to inhalation of environmental pollutants, contributing to development or exacerbation of inflammation [4]–[6]. That one of the main cellular sensors of hydrocarbons is now emerging as a regulator of innate immune responses may therefore be of particular importance. In addition, the role of AhR in inflammation does not seem to be restricted to effects of inhaled man-made chemicals.

There seems to be a considerable cross-talk between AhR-signaling and the nuclear factor-κB (NF-κB) family of transcription factors [7],[8]. The classical NF-κB-pathway typically characterized by the p65/p50 dimer is central in regulation of inflammatory responses through binding to κB-sites in the promoter region of a variety of pro-inflammatory genes, including several cytokines and chemokines [7],[8]. In lung cells, TCDD exposure increased NF-κB activity and IL-6 expression [9]. TCDD also induced IL-1β, IL-6 and CXCL8 (IL-8) through AhR-mediated activation of NF-κB in rheumathoid arthritis [10]. Furthermore, Vogel and colleagues [11],[12] showed that TCDD induced dimerization of AhR and RelB of the alternative NF-κB pathway and up-regulation of CXCL8 through a novel RelB/AhR response element (RelBAHRE) in macrophages and breast cancer cells [12]. Unligated AhR has been found to dimerize with p65 and activate κB-sites in the IL-6 and c-*myc* promoters [9],[13], and overexpression of constitutively activated AhR was associated with inflammatory skin lesions in mice [14]. In addition, the archetypical PAH benzo[*a*]pyrene (B[*a*]P) appeared to induce CXCL8 expression through binding of AhR to consensus XRE sites in the CXCL8 promoter [15].

In stark contrast, AhR-deficient mice displayed increased NF-κB activity and inflammation in the lungs after inhalation of lipopolysaccharide (LPS), cigarette smoke as well as instillation of crystalline silica [16],[17]. AhR knock-down also seems to cause hypersensitivity towards systemic inflammation in mice exposed to LPS by intraperitoneal injection [18],[19], and AhR deprivation increases LPS-induced cytokine responses in various cell types [18],[20],[21]. In addition, AhR-agonists such as TCDD and β-naphthoflavone (BNF) suppressed NF-κB signaling and cytokine responses induced by TNF-α, LPS and crystalline silica [17],[21],[22]. Thus, AhR seems to elicit both pro- and anti-inflammatory functions and both enhance and suppress NF-κB activity in the lung and other tissues.

We have previously found that 1-nitropyrene (1-NP), a prominent PAH in diesel exhaust, induced a cytokine/chemokine expression profile in human bronchial epithelial cells (BEAS-2B) characterized by high levels of CXCL8, whereas its amine metabolite 1-aminopyrene (1-AP) induced a different response characterized by increased CCL5 (RANTES) [23],[24]. CXCL8 is a potent neutrophil attractant associated with innate immune responses [25],[26], while CCL5 activates and attracts eosinophils commonly involved in allergic reactions [27]. CXCL8 may also contribute to tumor growth [28]–[30], while CCL5 may induce anti-tumor responses [31]–[33]. In addition to their differential biological properties, expression of CXCL8 and CCL5 also appear to be regulated differentially in airway epithelial cells [34],[35]. The effects of 1-NP on CXCL8 seems to involve ROS-formation and signaling through the TACE/TGF-α/EGFR-cascade [36]–[38], while the mechanisms of 1-AP induced CCL5 are less clear. However, the chemokine responses induced by both compounds seems at least partly to involve activation of β2-adrenergic receptors [39] (as well as unpublished results from our lab). Due to their differential effects, the 1-NP-induced CXCL8 *versus* 1-AP-induced CCL5 represented an intriguing model for studying the impact of AhR and Arnt on pro-inflammatory responses.

In the present work we have explored the roles of AhR and Arnt in the regulation of CXCL8 and CCL5 with emphasis on the involvement of the classical and alternative NF-κB pathway in BEAS-2B cells. Cells were exposed to 1-NP and 1-AP as well as polyinosinic-polycytidylic acid (Poly I:C), a synthetic double-stranded RNA analogue and Toll-like receptor 3 (TLR3) agonist known to induce NF-κB signaling and both CXCL8 and CCL5 responses in BEAS-2B cells [38]. The results suggest that the constitutive (endogenous) activity of unligated AhR and Arnt differentially suppress CXCL8 and CCL5 responses, and we propose an explanation for how AhR may both induce and suppress NF-κB signaling, even within the same cell type. The results also suggest that anti-inflammatory effects of AhR necessarily extend beyond interactions with the NF-kB pathway.

## Results

### Role of p65 in CXCL8 and CCL5 regulation in 1-NP- or 1-AP-exposed BEAS-2B cells

**Figure 1:**
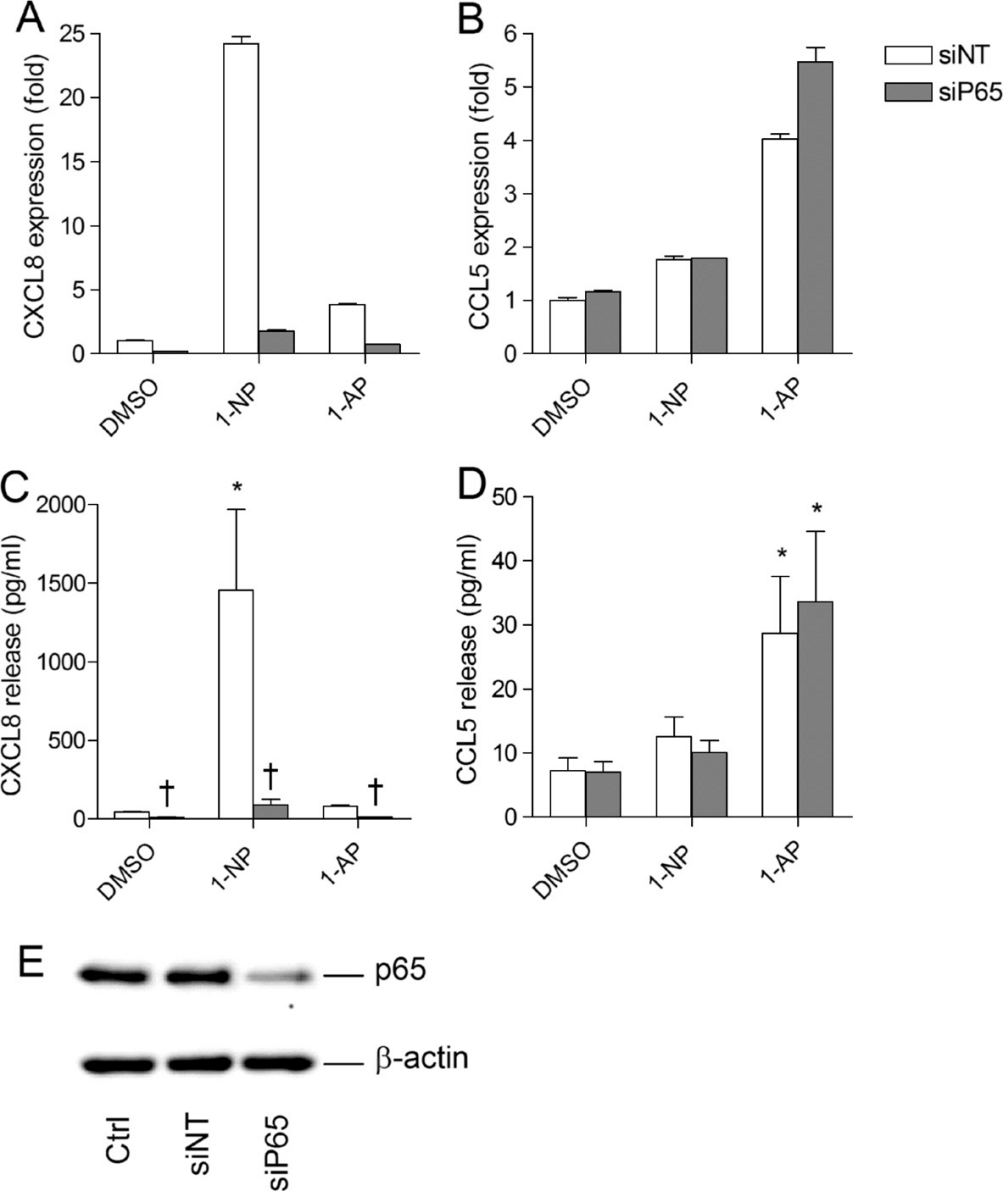
**P65 is required for CXCL8 but not CCL5 responses in 1-NP-or 1-AP-exposed BEAS-2B cells.** Cells were transfected with siRNA against p65 (siP65) or non-targeting control siRNA (siNT), and exposed to 20 μM 1-NP, 1-AP or vehicle (DMSO) alone. CXCL8 **(A)** and CCL5 **(B)** gene expression were measured after 6 h by real-time PCR. CXCL8 **(C)** and CCL5 **(D)** protein levels in the medium were measured by ELISA after 18 h exposure as described under “Materials and methods”. Efficiency of transfection was assessed by Western blotting **(E)**. The results are expressed as mean ± SEM (A/B: n = 1 (triplicate determinations); C/D: n ≥ 3; E: representative blot, n = 3). *Significantly different from unexposed controls; †Significantly different from cells transfected with non-targeting siRNA.

We have previously shown that 1-NP induces CXCL8, while 1-AP induces CCL5 in BEAS-2B cells [23],[24]. As a first step, we explored the involvement of the classical NF-κB pathways in the 1-NP- and 1-AP-induced chemokine responses by transfecting the cells with siRNA against p65 or non-targeting control siRNA. The classical NF-κB pathway is generally considered necessary for transcription of CXCL8 [40]. In line with this, we found that p65 silencing completely blocked both basal and induced CXCL8 responses (Figure 1A and C). In contrast, p65 siRNA had little impact on, or rather increased, the 1-AP-induced CCL5 response (Figure 1B and D). The protein level of p65 was markedly down-regulated in cells treated with the p65 siRNAs, confirming the efficiency of the transfection (Figure 1E). The above results suggest that the classical NF-κB pathway is needed for the CXCL8 response, but not for CCL5 in our cell model.

### Role of AhR and Arnt in CXCL8 and CCL5 regulation in 1-NP- or 1-AP-exposed BEAS-2B cells

**Figure 2:**
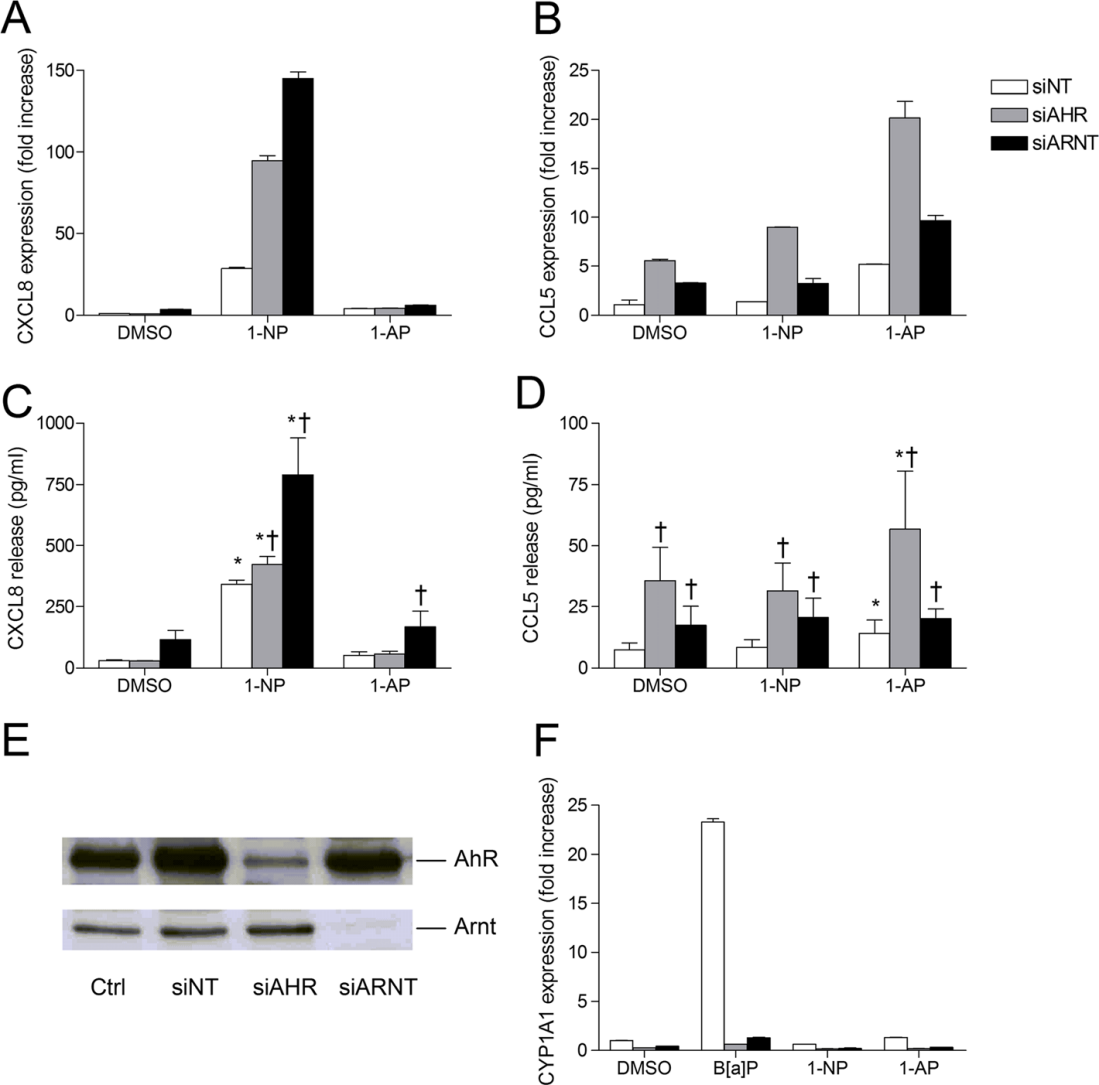
**AhR and Arnt differentially regulate CXCL8 and CCL5 responses in 1-NP-or 1-AP-exposed BEAS-2B cells.** Cells were transfected with siRNA against AhR (siAHR) and Arnt (siARNT) or non-targeting control siRNA (siNT), and exposed to 20 μM 1-NP, 1-AP or vehicle (DMSO) alone. CXCL8 **(A)** and CCL5 **(B)** gene expression were measured after 6 h by real-time PCR. CXCL8 **(C)** and CCL5 **(D)** protein levels in the medium were measured by ELISA after 18 h exposure as described under “Materials and methods”. Efficiency of transfection was assessed by Western blotting **(E)** as well as expression of CYP1A1 after 6 h exposure to 20 μM B[a]P, 1-NP and 1-AP, by real-time PCR **(F)**. The results are expressed as mean ± SEM (A/B/F: n = 1 (triplicate determinations); C/D: n ≥ 3; E: representative blots, n ≥ 3). *Significantly different from unexposed controls; †Significantly different from cells transfected with non-targeting siRNA.

As a next step we explored the roles of AhR and Arnt in the regulation of 1-NP-induced CXCL8 and 1-AP-induced CCL5. Thus, BEAS-2B cells were transfected with siRNA against AhR, Arnt or non-targeting control siRNA prior to exposure. Silencing of both AhR and Arnt increased 1-NP-induced CXCL8 mRNA expression after 6 h exposure and protein release after 18 h exposure (Figure 2A and C), with a markedly higher response in cells transfected with Arnt siRNA. However, only silencing of AhR induced a strong increase in both basal and induced CCL5 (Figure 2B and D). In comparison, targeting Arnt resulted in a more moderate increase in CCL5 that seemed restricted to basal and not induced levels, as 1-AP failed to increase CCL5 release in Arnt-depleted cells (Figure 2D). Notably, AhR silencing increased basal levels of CCL5 but not CXCL8, indicating that AhR regulated the two chemokines differentially. In comparison Arnt silencing seemed to induce an increase in basal levels of both CXCL8 and CCL5. This was confirmed by a more detailed examination of the effects on basal chemokine levels (Additional file 1: Figure S1, online supplementary materials). The efficiency of the siRNA transfection was verified by Western blotting, showing depletion of AhR and Arnt by their respective siRNAs (Figure 2E). In addition, AhR and Arnt silencing also reduced basal CYP1A1 expression and blocked B[*a*]P-dependent induction of CYP1A1 (Figure 2F), which further confirmed the efficiency of the siRNA transfections.

**Figure 3:**
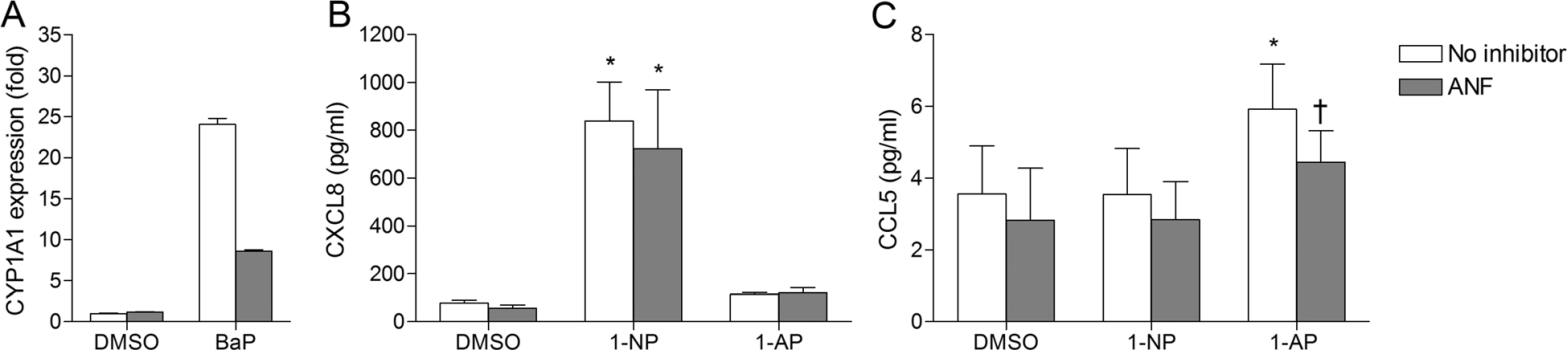
**Blocking of AhR ligand binding does not increase CXCL8 and CCL5 responses in BEAS-2B cells.** Cells were incubated with 0.5 μM of the AhR antagonist α-naphtoflavone (ANF) for 30 min prior to exposure for 18 h to 20 μM B[*a*]P, 1-NP, 1-AP or vehicle (DMSO) alone. CYP1A1 **(A)** levels were measured by real-time PCR, while CXCL8 **(B)** and CCL5 **(C)** levels in the medium were measured by ELISA as described under “Materials and methods”. The results are expressed as mean ± SEM (A: n = 1 (triplicate determinations); B/C: n = 5). *Significantly different from unexposed controls; †Significant effect of ANF.

Having shown that AhR and Arnt depletion enhanced CXCL8 and CCL5 responses, we next explored whether blocking the PAH-binding site of AhR would result in similar effects. Indeed, both 1-NP and 1-AP seem to have the ability to bind and activate AhR in other cells types [41],[42], although they did not induce CYP1A1 in the BEAS-2B cells (Figure 2F). Thus, to investigate whether the suppressive effects of AhR and Arnt on CXCL8 and CCL5 were related to ligand-mediated activation, BEAS-2B cells were treated with the AhR antagonist α-naphthoflavone (ANF), prior to PAH exposure. Pretreatment with 0.5 μM of ANF reduced B[*a*]P-induced CYP1A1 induction by more than 50%, confirming the efficiency of the antagonist (Figure 3A). However, in contrast to the enhanced chemokine responses observed by siRNA transfection (Figure 2), blocking the ligand-binding site of AhR with ANF did not affect CXCL8 levels (Figure 3B), and rather decreased 1-AP-induced CCL5 (Figure 3C).

The above results suggest that AhR acts as a suppressor of both 1-NP-induced CXCL8 and 1-AP-induced CCL5, while Arnt more selectively suppresses 1-NP-induced CXCL8 responses in BEAS-2B cells. These effects seemed to be independent of ligand-mediated activation of AhR, and may therefore rather be due to basal or constitutive receptor activity. Moreover, since CCL5 seemed to be regulated independently of p65 (Figure 1), suppression of CCL5 by AhR was most likely mediated through NF-κB-independent mechanisms.

### Induction of NF-κB activity by 1-NP and TNF-α, and the roles of AhR and Arnt

**Figure 4:**
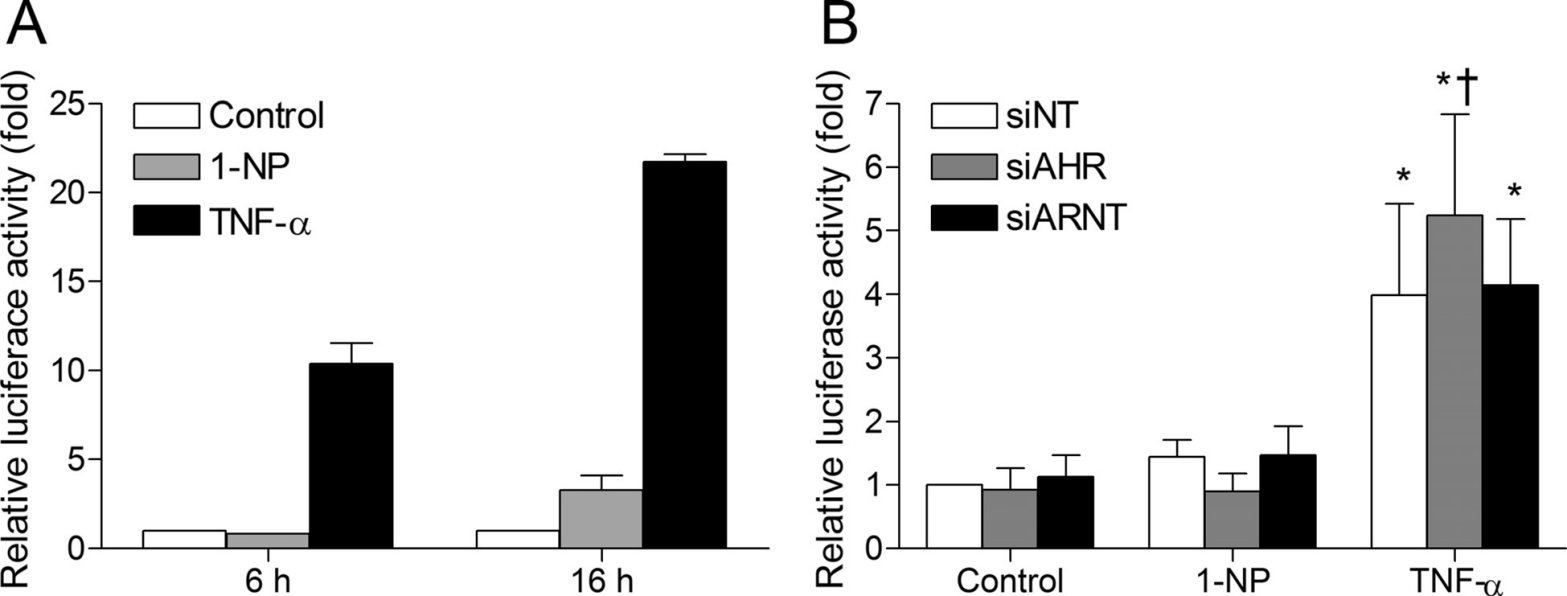
**AhR silencing increased TNF-α-,but not 1-NP-induced p50/p65 reporter gene expression in BEAS-2B cells.** Cells were transfected with a NF-κB-luciferase (p50/p65) reporter gene, and exposed to 1-NP or TNF-α for 6 or 16 h **(A)**, and assayed for luciferase activity as described under “Materials and methods”. Cells transfected siRNAs against AhR (siAHR), Arnt (siARNT) or non-targeting control siRNA (siNT) were further transfected with NF-κB-luciferase promoter and assayed for luciferase activity after a 16 h exposure to 1-NP (20 μM) or TNF-α (50 ng/ml) **(B)**. The results are expressed as mean ± SEM (A: n = 2; B: n = 5). *Significantly different from unexposed controls; †Significantly different from cells transfected with non-targeting siRNA.

Since basal and 1-NP-induced CXCL8 seemed dependent on p65 expression, we next investigated the effect of 1-NP on activation of NF-κB using a luciferase assay for p65 activity. In an initial screen, the pro-inflammatory cytokine TNF-α used as positive control appeared to induce a strong increase in NF-κB-driven luciferase activity after both 6 and 16 h exposure (Figure 4A). In comparison, 1-NP did not affect luciferase activity at the early time point and only induced a moderate increase after 16 h (Figure 4A). However, this effect was not statistically significant in the next set of experiments (Figure 4B). Moreover, no effect on IκB-degradation or phosphorylation of p-p65 were observed after 2 or 4 h exposure to 1-NP or 1-AP (Additional file 2: Figure S2, online supplementary material). Considering that 1-NP induced a marked increase in CXCL8 mRNA expression already after 4 h exposure (Figures 1A and 2A), it does not seem that 1-NP mediated the CXCL8 response through activation of the classical NF-κB pathway. Thus, p65 most likely played a strictly permissive role in 1-NP-induced CXCL8 (Figure 1), and that the observed effect of p65 silencing was due to attenuation of the basal transcription factor activity. In line with this, p65 depletion also reduced CXCL8 levels in controls and 1-AP-exposed cells (Figure 1).

To assess any possible influence of AhR and Arnt on p65 activity, we measured p65-induced luciferase activity in AhR- or Arnt-depleted BEAS-2B cells after 16 h exposure to 1-NP or TNF-α. AhR silencing by siRNA did not affect basal or 1-NP-induced p65 activity significantly (Figure 4B). If anything, the low-level induction of p65 activity by 1-NP was rather reduced. Thus, it seems that not only 1-AP-induced CCL5, but also the 1-NP-induced CXCL8 response was suppressed by AhR through mechanisms other than interference with the classical NF-κB pathway. However, AhR silencing resulted in a moderate, but statistically significant increase in TNF-α-induced p65 activity (Figure 4B), suggesting that the constitutive activity of AhR modestly suppresses the p65 signaling induced by stronger activators of the classical NF-κB pathway. Cells transfected with Arnt siRNA did not display any changes in luciferase activity as compared to non-targeting control siRNA (Figure 4B).

### Role of AhR and Arnt in p65 and CXCL8/CCL5 regulation in Poly I:C-exposed BEAS-2B cells

**Figure 5:**
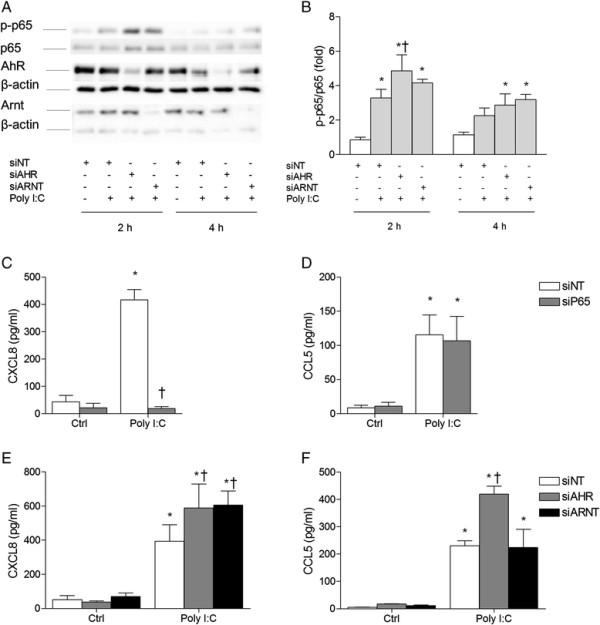
**AhR and Arnt differentially regulate CXCL8 and CCL5 responses as well as p65 phosphorylation in Poly I:C-exposed BEAS-2B cells.** Cells were transfected with siRNA against p65 (siP65), AhR (siAHR), Arnt (siARNT) or non-targeting control siRNA (siNT), and exposed to 10 μg/ml Poly I:C. Intracellular protein levels of total and phospho-p65 (Ser536) as well as AhR and Arnt were detected by Western blotting after 2 and 4 h exposure, as described under “Materials and methods”. The figure displays representative blots **(A)**, as well as relative changes in phospho-p65 compared to total p65 quantified by densitometric analysis of the Western blots **(B)**. CXCL8 **(C and E)** and CCL5 **(D and F)** protein levels in the medium were measured by ELISA after 18 h exposure, as described under “Materials and methods”. The results are expressed as mean ± SEM (n ≥ 3). *Significantly different from unexposed controls; †Significantly different from cells transfected with non-targeting siRNA.

To examine if the chemokine regulation by AhR and Arnt were of a more general nature, we continued by exploring their effects in cells exposed to the Toll-like receptor 3 (TLR3)-ligand Poly I:C. In agreement with previous reports [43],[44], Poly I:C induced strong increases in both CXCL8 and CCL5 along with activation of the classical NF-κB pathway, as evidenced by the degradation of IκB and increased phosphorylation of p65 at serine 536 (Additional file 2: Figure S2, online supplementary materials). This allowed for assessing the effects of AhR and Arnt on CXCL8 and CCL5 responses induced by a single compound without known affinity for the AhR or CYP-enzymes, that unlike 1-NP and 1-AP, also activated the classical NF-κB pathway. We thus examined the effects of targeting p65, AhR and Arnt by siRNA in Poly I:C-exposed cells. In line with the observed increase in TNF-α-induced p65 activity by AhR-silencing (Figure 4B), we found that depletion of AhR resulted in increased Poly I:C-induced phosphorylation of p65 at serine 536 (Ser536) in BEAS-2B cells at 2 h, while Arnt depletion did not cause any significant effects (Figure 5A and B). This strengthens the notion that AhR suppresses activation of p65-signaling induced by strong inducers of the classical NF-κB pathway. As in PAH-exposed cells (Figure 1), we again observed that CXCL8, but not CCL5, was dependent on p65 (Figure 5C and D). In further agreement with the data obtained by 1-NP and 1-AP exposure (Figure 2), silencing of both AhR and Arnt increased CXCL8 responses in Poly I:C-exposed BEAS-2B cells (Figure 5E), while only AhR silencing augmented CCL5 (Figure 5F). The effects obtained by the custom made siRNA against AhR were confirmed by use of commercially available siRNA (Additional file 3: Figure S3, online supplementary materials). This fortifies the observations made by PAH treatment and suggests that AhR and Arnt may play a general role in the regulation of CXCL8 and CCL5 in BEAS-2B cells, independently of exposure. The results further suggest that AhR may interfere with phosphorylation of p65 at Ser536 which is considered important for transactivation of p65 and transcription of CXCL8 [45],[46]. Moreover, as CCL5 was not affected by p65 silencing, AhR necessarily also regulated Poly I:C-induced CCL5 expression through interactions with other pathways.

### Interaction with CXCL8 and CCL5 promoters and subcellular localization of p65, AhR and Arnt in Poly I:C-exposed BEAS-2B cells

**Figure 6:**
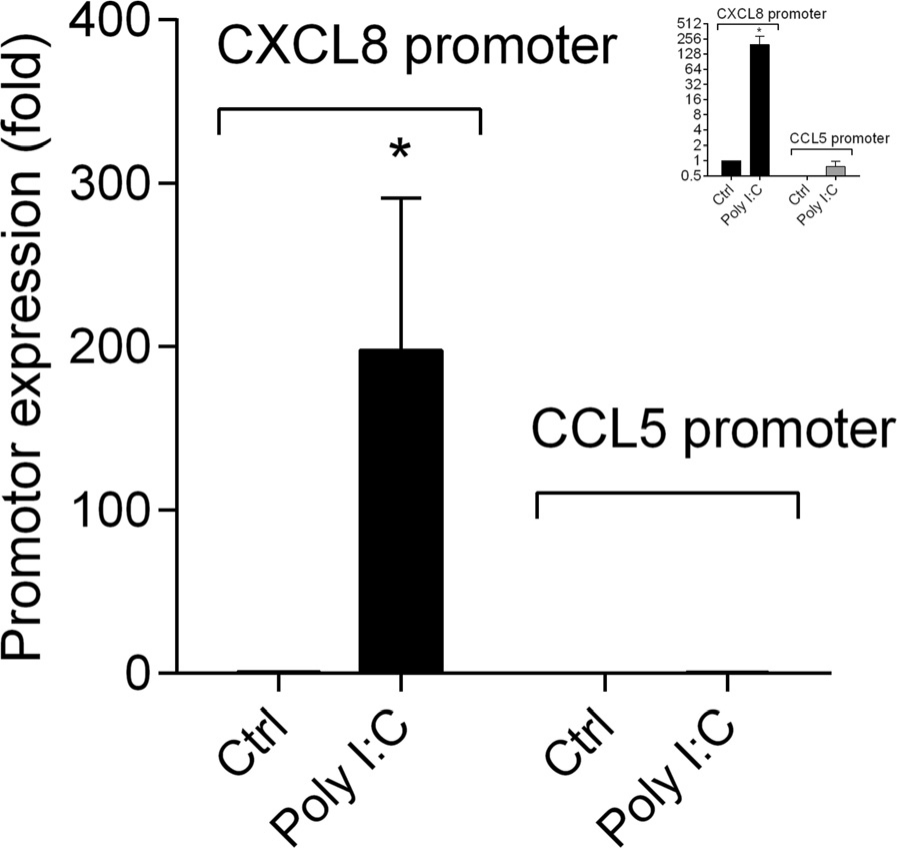
**Interaction between p65 and the CXCL8 and CCL5 promoters in Poly I:C exposed BEAS-2B cells.** Cells were exposed to 10 μg/ml Poly I:C for 3 h. Interaction between p65 and the NF-κB responsive regions of the CXCL8 and CCL5 promoters were assessed by ChIP assay as described under Materials and methods”. Results are expressed as fold increase compared to p65 binding to the CXCL8 promoter after adjustment for the input control (GAPDH). The results are expressed as mean ± SEM ( n = 6). The insert figure shows the same data set on a log-scale. *Significantly different from unexposed controls.

For a more complete understanding of the roles of p65, AhR and Arnt in CXCL8 and CCL5 regulation, we also assessed to what extent they interacted directly with the NF-κB-response element containing regions of the CXCL8 and CCL5 promoters when cells were exposed to ligands. In line with the above results, ChIP assay revealed that Poly I:C treatment induced a strong increase in p65 binding to the CXCL8 promoter, but not to the CCL5 promoter (Figure 6). Moreover, we were unable to detect binding of AhR and Arnt to the CXCL8 and CCL5 promoters by ChIP (data not shown). Thus, neither AhR nor Arnt appeared to suppress chemokine responses by interfering directly the NF-κB responsive regions of the CXCL8 and CCL5 promoters. Furthermore, we also assessed the subcellular localization of p65, AhR and Arnt by immunocytochemistry. In control cells, p65 was primarily located in the cytosol, and translocated into the nucleus upon exposure to Poly I:C and partly also when exposed to 1-NP (Additional file 4: Figure S4, online supplementary materials). AhR and Arnt on the other hand, appeared to be located both in the cytosol and in nucleus of resting cells, and no apparent change were observed in the sub-cellular localization upon exposure (Additional file 4: Figure S4, online supplementary materials). This is in line with the observation that AhR signaling was not activated by the exposure.

### Role of RelB in AhR and Arnt-mediated regulation of CXCL8 and CCL5

**Figure 7:**
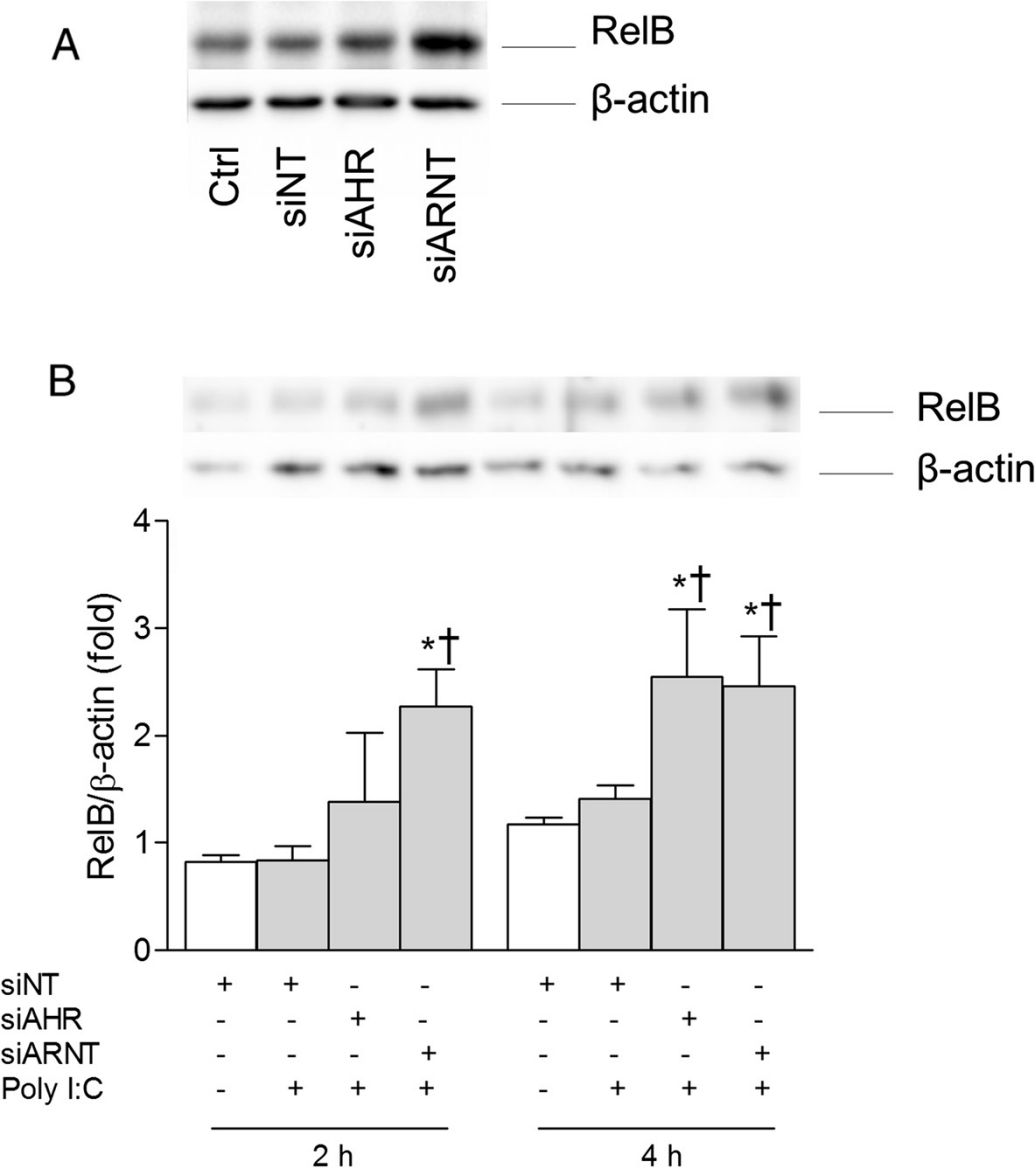
**Depletion of AhR and Arnt increases RelB levels in Poly I:C**-**exposed BEAS-2B cells.** Cells were transfected with siRNA against AhR (siAHR), Arnt (siARNT) or non-targeting control siRNA (siNT), and exposed to 10 μg/ml Poly I:C for 2 and 4 h. Intracellular protein levels of RelB and β-actin were detected by Western blotting as described under “Materials and methods”. The figure displays representative blots of RelB levels in unexposed cells **(A)** and Poly I:C-exposed cells **(B)**. The graph depicts relative changes in RelB **(B)** compared to β-actin quantified by densitometric analysis of the Western blots. The results are expressed as mean ± SEM (n = 3). *Significantly different from unexposed controls; †Significantly different from cells transfected with non-targeting siRNA.

RelB of the alternative (non-canonical) NF-κB pathway is a well-known suppressor of the classical NF-κB pathway and inflammation that itself is under transcriptional regulation by p65 [47]–[49]. Therefore, if AhR or Arnt silencing enhances p65-signaling, RelB should be increased in a similar manner as CXCL8. Conversely, it has been proposed that the anti-inflammatory role of AhR is related to stabilization of RelB, resulting in a more rapid degradation of RelB in AhR-depleted cells or tissues exposed to cigarette smoke [16],[20]. We first examined the impact of AhR and Arnt silencing on RelB expression. The results show that basal RelB levels were markedly increased in cells transfected with Arnt, but not AhR siRNA (Figure 7A). RelB levels were also increased in Arnt-depleted cells exposed to Poly I:C for 2 and 4 h (Figure 7B). In contrast, AhR silencing first increased RelB levels after 4 h exposure. Therefore, as observed with CXCL8, Arnt seemed to suppress both basal and induced RelB while AhR more selectively suppressed only induced RelB levels. The latter is consistent with the observation that AhR only suppressed induced and not basal p65 activity. However, the Arnt-mediated suppression of RelB suggests that Arnt may interfere with p65-signaling despite its lack of effect on p65 activation (Figures 4 and 5). Furthermore, the results suggest that neither AhR nor Arnt suppressed chemokine responses by stabilizing RelB in Poly I:C-exposed BEAS-2B cells.

**Figure 8:**
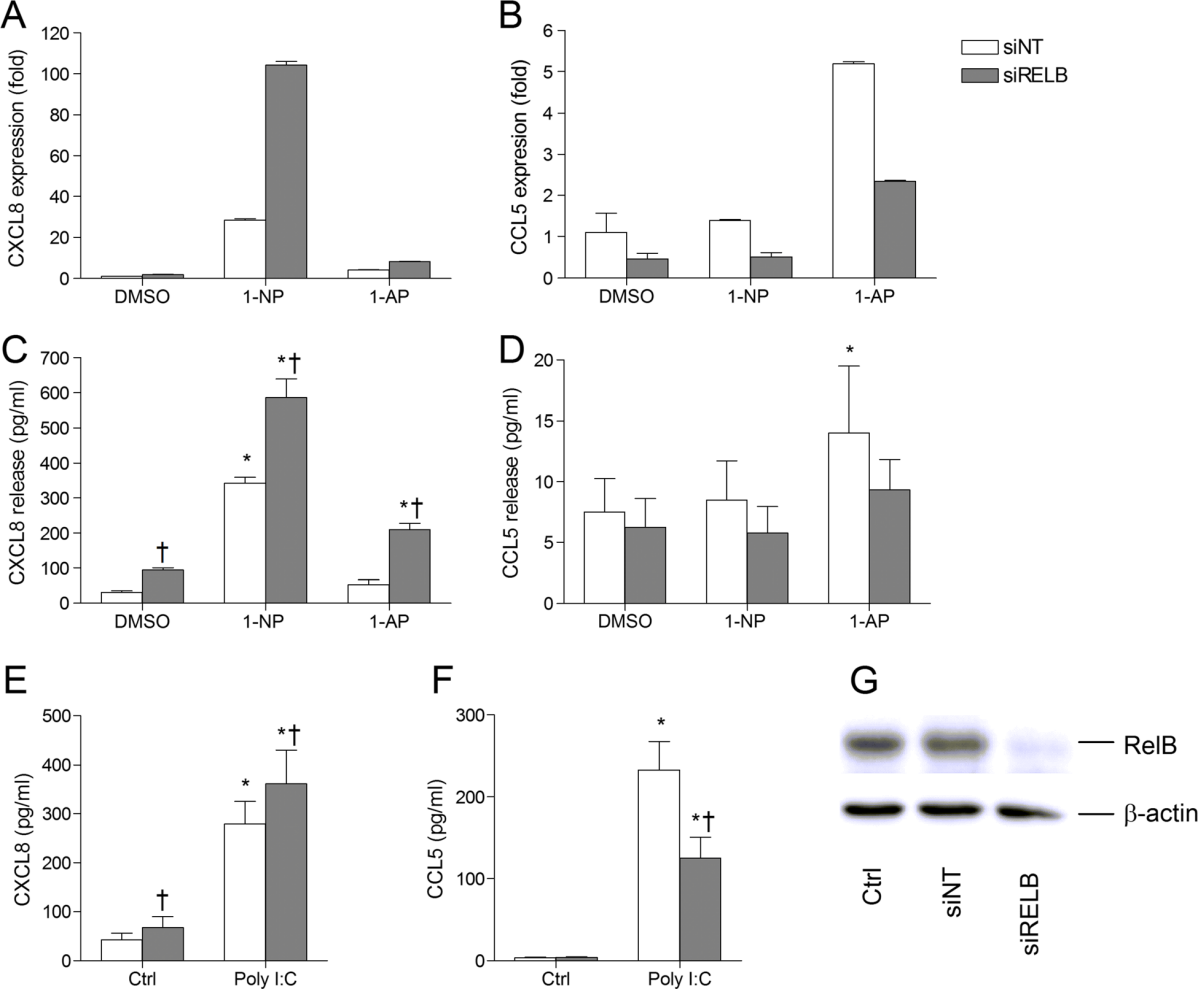
**RelB suppresses CXCL8, but not CCL5, in BEAS-2B cells.** Cells were transfected with siRNA against RelB (siRELB) or non-targeting control siRNA (siNT). Transfected cells were exposed to 20 μM 1-NP, 1-AP, or vehicle (DMSO) alone **(A-D)** or 10 μg/ml Poly I:C or vehicle (water) alone **(E and F)**. CXCL8 **(A)** and CCL5 **(B)** gene expression were measured after 6 h by real-time PCR. CXCL8 (C and E) and CCL5 **(D and F)** protein levels in the medium were measured by ELISA after 18 h exposure as described under “Materials and methods”. Efficiency of transfection was assessed by Western blotting **(G)**. The results are expressed as mean ± SEM (A/B: n = 1 (triplicate determinations); **C-F**: n ≥ 3; G: representative blot, n = 3) *Significantly different from unexposed controls; †Significantly different from cells transfected with non-targeting siRNA.

Notably, RelB has also been implicated in AhR- or Arnt-mediated transcription regulation in various ways [11],[12],[16],[20],[49]. So, to further explore whether RelB could be involved in AhR- or Arnt-mediated chemokine suppression, we then examined the effect of RelB silencing on chemokine responses in 1-NP-, 1-AP- or Poly I:C-exposed BEAS-2B cells. RelB depletion by siRNA increased CXCL8 responses (Figure 8A,C and E) but reduced CCL5 responses (Figure 8B,D and F). Thus, the effects of RelB partly resembled that of Arnt (Figures 2 and 5), by suppressing both basal and induced CXCL8 and not CCL5. However, in contrast to Arnt, RelB seemed to be required for CCL5 responses. The observation that AhR in addition to suppressing CCL5 selectively suppressed induced, but not basal CXCL8 levels (Figures 2 and 5), further suggests that AhR did not mediate its anti-inflammatory effects through RelB.

In contrast to the effects obtained by RelB siRNA (Figure 8), silencing its binding-partner in the alternative NF-κB pathways, p100/p52, attenuated both CXCL8 and CCL5 responses (Additional file 5: Figure S5A, online supplementary materials). Moreover, neither 1-NP, 1-AP or Poly I:C induced degradation of p100 to p52 (Additional file 5: Figure S5B, online supplementary materials), the hallmark of alternative NF-κB activation [50]. This suggests that the RelB-mediated suppression of CXCL8 (Figure 8) was independent of the alternative NF-κB pathway. However, it is possible that the RelB-dependency of CCL5 (Figure 8) may be related to some permissive role of the alternative NF-κB pathway.

### Activation of AhR by BNF suppresses Poly I:C-induced CXCL8 and CCL5

**Figure 9:**
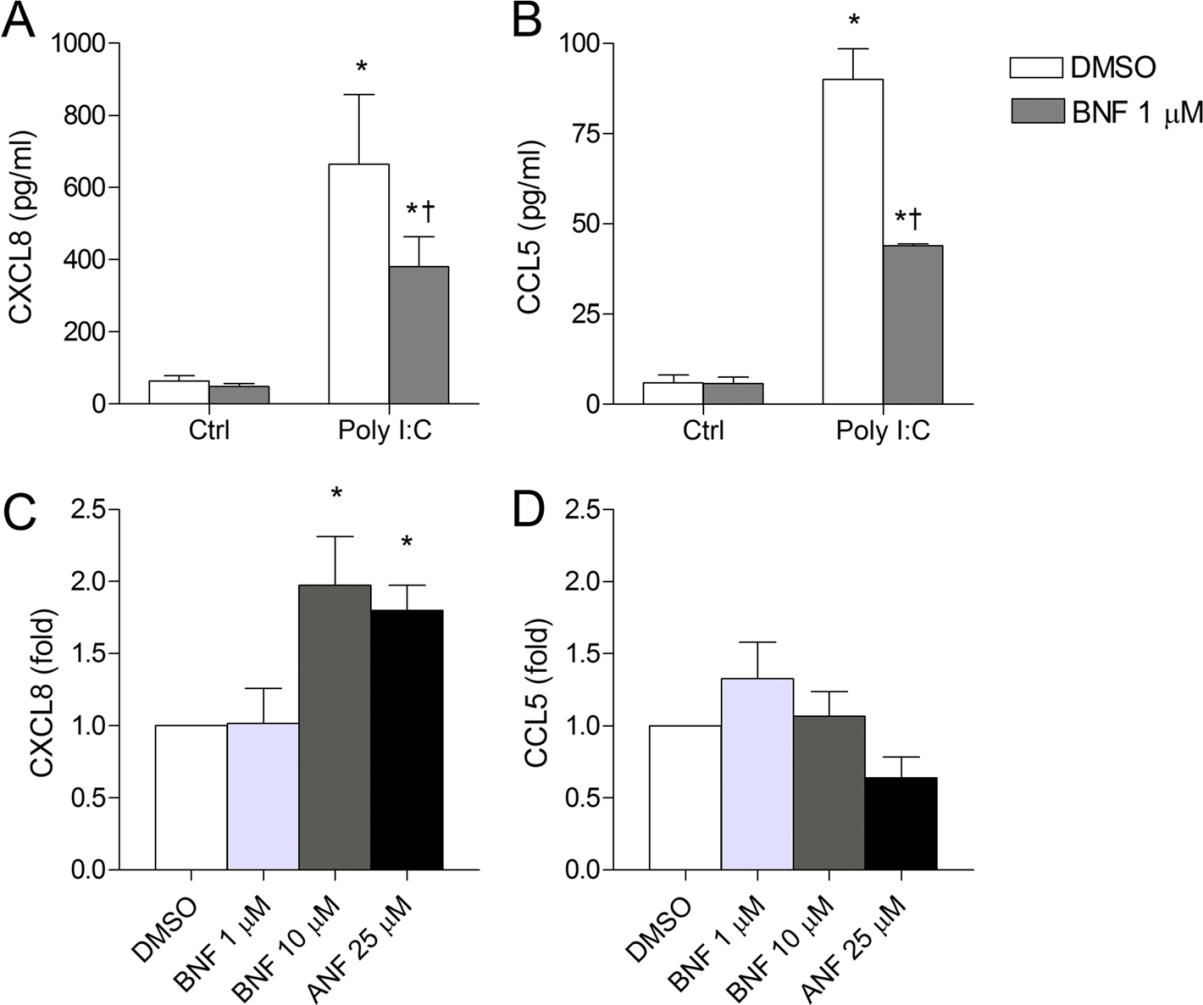
**AhR-activation suppresses Poly I:C-induced CXCL8 and CCL5 responses**, **but stimulate CXCL8 responses alone in BEAS-2B cells.** Cells were incubated with 1 μM of the AhR agonist β-naphtoflavone (BNF) for 30 min prior to exposure to 10 μg/ml Poly I:C for 18 h **(A and B)**, or exposed to high concentrations of ANF or BNF alone for 18 h **(C and D)**. CXCL8 **(A andC)** and CCL5 **(B and D)** levels in the medium were measured by ELISA as described under “Materials and methods”. The results are expressed as mean ± SEM (n = 3). *Significant increase induced by Poly I:C; †Significant reduction induced by BNF.

Our present results suggest that the constitutive activity of unligated AhR and Arnt regulate chemokine responses through differential mechanisms. As a final step, we also explored how ligand-mediated activation of AhR-signaling would affect CXCL8 and CCL5 regulation. It has been reported that the non-toxic AhR agonist β-naphthoflavone (BNF) may suppress TNF-α-induced NF-κB activation and LPS-induced cytokine responses [21],[22]. Thus we examined the effect of BNF on Poly I:C-induced CXCL8 and CCL5 in BEAS-2B cells. Pre-incubation with 1 μM BNF for 30 min prior to Poly I:C exposure for 18 h, attenuated both Poly I:C-induced CXCL8 and CCL5 (Figure 9A and B). This effect seem to extend to other cell types, as BNF suppressed Poly I:C-induced chemokine responses in A549 human alveolar type-II-like epithelial carcinoma cells and THP-1 human leukemia monocytes (Additional file 6: Figure S6, online supplementary materials). Of notice, Poly I:C appeared unable to induce CXCL8-responses in the A549 cells. We also observed that pre-incubation with another well-known AhR-agonist, B[*a*]P, suppressed both CXCL8 and CCL5 responses in BEAS-2B cells exposed to Poly I:C or LPS (Additional file 7: Figure S7, online supplementary materials). In contrast, treatment with high concentrations of BNF and ANF (10 and 25 μM, respectively) alone, induced moderate two-fold increases in CXCL8 but not CCL5 (Figure 9C and D). Therefore it seems that not only the constitutive activity of unligated AhR, but also ligand-mediated activation of the AhR may suppress pro-inflammatory responses in BEAS-2B cells. These results also show that activation of AhR by external ligands increase the suppression of cytokine/chemokine responses compared to the effects of unligated, constitutively active AhR. However, in absence of other, stronger pro-inflammatory stimuli, AhR-ligands elicited a moderat pro-inflammatory effect in BEAS-2B cells when given at sufficiently high concentrations.

## Discussion

**Figure 10:**
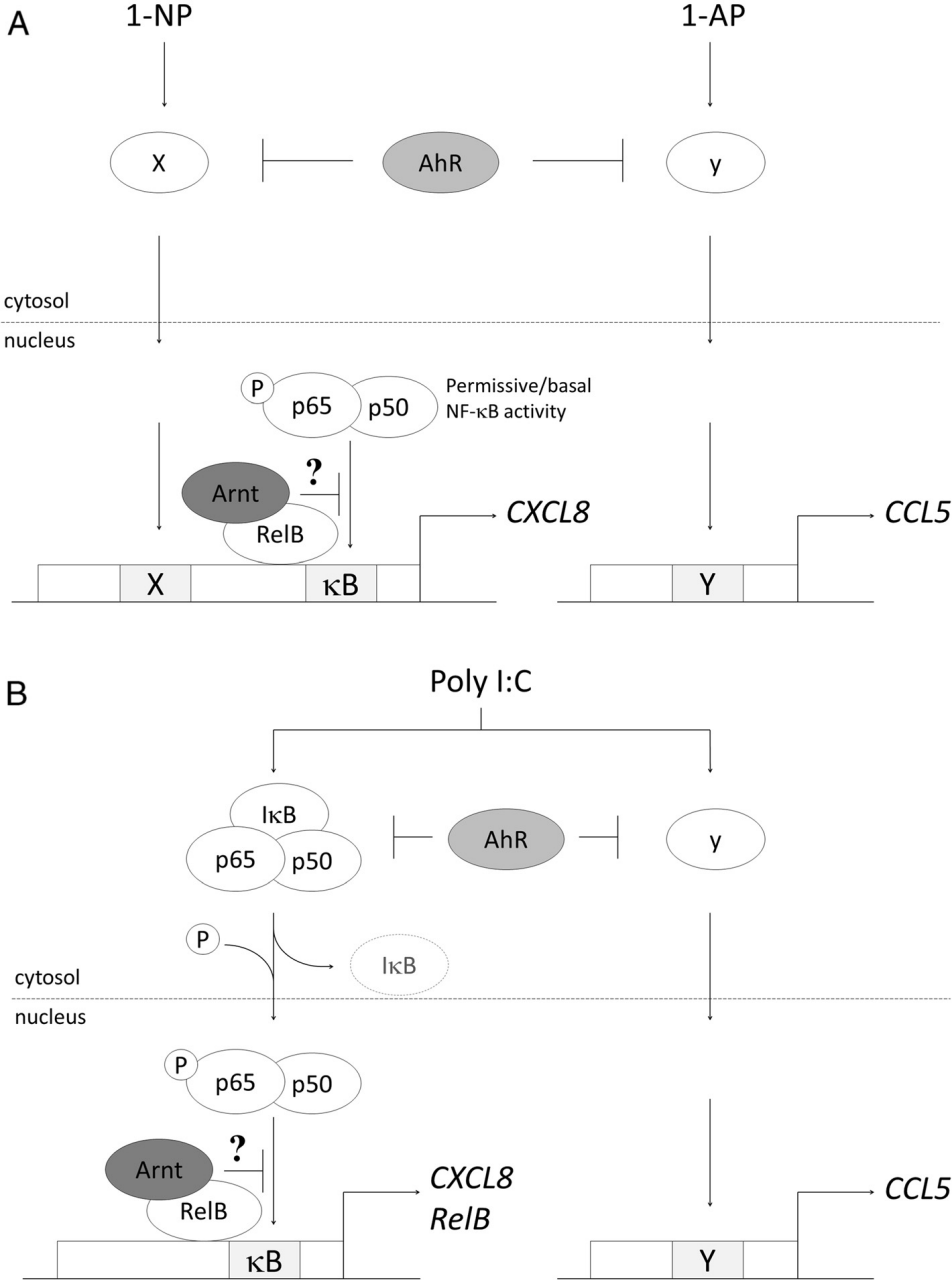
**The constitutive activity of unligated AhR and Arnt differentially regulate CXCL8 and CCL5 in BEAS-2B cells.** The model summarizes the possible pathways discussed for AhR- and Arnt-mediated regulation of CXCL8 and CCL5 in BEAS-2B cells exposed to 1-NP and 1-AP **(A)** or Poly I:C **(B)**. “x/X” and “y/Y” represents hitherto unidentified signaling pathways/response elements involved in regulation of CXCL8 and CCL5, respectively. The positioning of the response elements in the CXCL8 or CCL5 promoters is not meant to be representative. Moreover, “y/Y” induced by 1-AP **(A)** and Poly I:C **(B)** are not necessarily identical and AhR may also affect other pathways involved in Poly I:C-induced CXCL8 in addition to NF-κB **(B)**. The suggested position and association of Arnt and RelB is partly based on findings reported by Wright and Duckett [49]. The role of AhR and Arnt in chemokine regulation may differ in other cell types.

In the present study we have compared the effects of AhR and Arnt depletion on the classical NF-κB pathway and expression of two differentially regulated chemokines, CXCL8 and CCL5, in bronchial epithelial BEAS-2B cells exposed to PAHs or the TLR3 agonist Poly I:C. The results showed that regulation of CXCL8, but not CCL5, was dependent on basal and/or induced p65 activity. AhR suppressed both CXCL8 and CCL5 responses in BEAS-2B cells by widely differing stimuli, while Arnt only suppressed CXCL8. Moreover, the AhR-antagonist ANF failed to reproduce the effects of AhR knock-down on CXCL8/CCL5 regulation. This suggests that AhR suppressed chemokine responses independently of Arnt, and the effects were most likely due to the constitutive activity of unligated AhR. AhR suppressed p65 activation induced by strong activators of the classical NF-κB pathway, such as TNF-α and Poly I:C, but did not affect basal p65 activity. In addition, since AhR also suppressed CCL5, as well as the p65-independent increase in CXCL8 by 1-NP (which were unable to enhance p65 activity), the anti-inflammatory effects of AhR were necessarily not restricted to interference with NF-κB-signaling. In contrast to AhR, Arnt seemed unable to suppress the onset of p65 activation. Nevertheless, Arnt may still interfere with p65 signaling through other mechanisms, possibly in collaboration with RelB, which is a well-known suppressor of p65 activity. A schematic presentation of the possible roles of AhR and Arnt in suppression of CXCL8 and CCL5 in BEAS-2B cells, as discussed below, is presented in Figure 10. In addition to the above, we also observed that potent-AhR agonists could suppress both CXCL8 and CCL5-responses by TLR-ligands, but given alone at sufficiently high concentrations, they elicited a moderate activation of CXCL8. Thus ligand-activated AhR appear to possess both pro- and anti-inflammatory effects in BEAS-2B cells.

In an elegant series of experiments Chen and colleagues [9] showed that AhR could induce IL-6 expression and NF-κB activity in BEAS-2B cells and H1355 human lung adenocarcinoma cells. Overexpression of AhR resulted in increased formation of AhR/p65-complexes that activated κB-sites in the IL-6 promoter, while TCDD-exposure activated the classical p65/p50-heterodimer [9]. In line with this, we observed that high concentrations of the AhR-ligands ANF and BNF induced a moderate increase in CXCL8, but not the p65-independent CCL5. However, in extension of the findings by Chen et al., [9] we further observed that AhR in the same cell type may also possess anti-inflammatory properties, and we propose that whether AhR act as suppressor or inducer of inflammation largely depends on the type and combination of stimuli. Importantly, our present results show that AhR depletion did not suppress basal p65 activity or CXCL8 levels in unexposed cells. However, NF-κB activation induced by TNF-α and Poly I:C was significantly enhanced. This may at least partly, be due to AhR-mediated suppression of p65-phosphorylation at Ser536, which is important to CXCL8 transcription by promoting coupling of p65 to the basal transcriptional machinery [45],[46],[51]. Phosphorylation of p65 at Ser536 is mediated by Inhibitor of κB Kinases (IKKs) [45],[51]. Recent findings suggest that AhR may interact directly with IKKs [52], providing a possible explanation for the observed effects of AhR knock-down on Poly I:C-induced Ser536 phosphorylation of p65. In addition, the study by Chen and co-workers [9] indicates that AhR/p65 may be a less efficient activator of κB-sites than p65/p50 (TCDD-exposure induced more than twice the NF-κB activity elicited by AhR-overexpression, despite almost identical binding of p65 to the IL-6 promoter). It is therefore conceivable that AhR/p65-dimerization may restrict the availability of free p65 levels, thus limiting formation of the more potent p65/p50-dimers when cells are exposed to strong, AhR-independent, activators of the classical NF-κB pathway such as Poly I:C, TNF-α or LPS. A recent publication by Vogel and colleagues [53] offers an interesting perspective to our current findings. In their study, they found that AhR was under transcriptional regulation by p65, and that LPS exposure induced AhR expression in human dendritic cells [53]. Thus, the suppressive effects of AhR on p65 activity may possibly be ascribed to a negative feedback mechanism. However, in contrast to these observations Poly I:C exposure did not induced AhR expression in BEAS-2B cells, but rather appeared to reduce the level of AhR. In any case, the scenario of AhR as a partial NF-κB agonist acting antagonistic in competition with stronger NF-kB activators may provide an explanation for how different studies have observed that AhR either induces or suppresses NF-κB signaling.

In contrast to AhR, Arnt did not seem to affect activation of the classical NF-κB pathway as assessed by p65-luciferace activity or p65 phosphorylation. Yet, Arnt depletion enhanced the expression of the NF-κB target gene RelB [47],[49] in both un-stimulated and Poly I:C-exposed BEAS-2B cells. In addition, we observed that Arnt silencing selectively enhanced the p65-dependent chemokine CXCL8 and not the p65-independent CCL5. It seems that although Arnt did not appear to suppress the activation of p65, it may possibly still interfere with p65 signaling through some other mechanisms. Of notice, we further observed that RelB itself suppressed CXCL8 and not CCL5, in parallel with Arnt. In addition to being transcriptionally regulated by p65/p50, RelB is also a well-known suppressor of the classical NF-κB pathway and inflammation that may represent a negative feed-back loop for NF-κB regulation [48],[49]. Interestingly, Wright and Duckett [49] showed that Arnt facilitated RelB binding to NF-κB responsive promoters preventing p65/p50-binding and suppressing NF-κB-mediated transcription in CD30-exposed T-cells (Karpas 299 cells). In coherence with our present findings, they also observed that Arnt silencing increased RelB levels in CD30-stimulated cells [49]. Whether Arnt facilitates RelB-mediated suppression of p65 signaling also in BEAS-2B cells is an intriguing scenario that requires further investigation. If this is the case, the reason Arnt depletion did not affect the NF-κB luciferase-assay is likely that the Arnt/RelB-binding domain of wild-type NF-κB responsive promoters was lacking in the luciferase reporter-gene promoter.

Others have shown that increased inflammation in lungs of LPS and cigarette smoke (CS)-exposed AhR^−/−^ mice were associated with a rapid loss of RelB [16]. Thus, the anti-inflammatory role of AhR has been suggested to be due to RelB-stabilization [20]. Our present data does not suggest that this is the case in BEAS-2B cells. Silencing of AhR enhanced RelB levels in BEAS-2B cells, although first after 4 h exposure to Poly I:C. This is consistent with the observation that AhR suppressed activation of p65 by Poly I:C and TNF-α, but not basal p65 activity. In addition, we also observed that AhR and RelB were differentially involved in regulation of CXCL8 and CCL5, which further suggest that AhR does not mediate its effects through RelB.

Notably, although transfection with AhR siRNA did not affect p65 activity in 1-NP-exposed cells, the AhR silencing increased 1-NP-induced CXCL8 without affecting basal CXCL8 levels. In addition, AhR (in contrast to Arnt) also suppressed the p65-independent CCL5 responses in the BEAS-2B cells. This suggests that suppression of the classical NF-κB pathway cannot be the only mechanism for the anti-inflammatory effects of the AhR, but that at least two additional pathways must be affected (Figure 9). In line with this, it has been reported that the activity of other pro-inflammatory transcription factors such as activator protein-1 (AP-1) and CCAAT-enhancer binding protein (C/EBP) may be affected by AhR [54],[55]. Therefore it is tempting to speculate that the differential suppression of CXCL8 and CCL5 by AhR and Arnt in BEAS-2B cells could be due suppression of a broader range of pro-inflammatory pathways by AhR, or through effects on post-transcriptional or translational processes.

The majority of the present work focused on the anti-inflammatory role of constitutive (unligated) AhR and Arnt activity. However, we also observed that the AhR-agonist BNF as well as B[*a*]P suppressed Poly I:C-induced CXCL8 or CCL5 responses. Thus, ligand-mediated activation increased chemokine suppression by AhR, beyond the effects of the constitutive activity of the unligated receptor. Similarly BNF and other AhR ligands have been reported to suppress LPS-induced cytokine expression and inflammation, *in vitro* and *in vivo*[18],[21]. Moreover, in a recent study TCDD was found to suppress silica-induced inflammation [17]. Considering that TCDD has been reported to induce p65/p50 through AhR [9] and that our present CCL5-responses were unaffected by p65 depletion (Figures 4 and 7), it seems unlikely that BNF and B[*a*]P attenuated Poly I:C-induced CXCL8 and CCL5 through suppression of NF-κB signaling in the BEAS-2B cells. However, whether unligated and ligand-activated (i.e. endogenously and exogenously activated) AhR suppress inflammation through similar pathways remains to be clarified.

The present study was performed in BEAS-2B cells, a common model of human bronchial epithelial cells which has been reported to exhibit the highest homology in gene expression pattern with primary lung cells as compared to other established lung cell lines [56]. As any result obtained with immortalized cell lines, generalizations should always be interpreted with some caution. However, several *in vivo* studies with AhR knock-out mice or in primary mouse cells support the notion that AhR suppresses induction of NF-κB activity and inflammatory reactions in different cells and tissues [16],[17],[19]–[21]. Moreover, in control experiments with A549 and THP-1 cells, we observed comparable inhibition of Poly I:C-induced chemokine responses by the AhR agonist BNF (Additional file 5: Figure S5, online supplementary materials), further suggesting that the present findings are not restricted to the BEAS-2B cell line. However, in a recent paper, Vogel and colleagues [57] observed that combined exposure with TCDD enhanced the expression of IL-6, IL-10, IL-22, IL-23, CXCL2 and CXCL3 in LPS-stimulated dendritic cells. Only LPS-induced DC-CK1 was suppressed by TCDD exposure, while IL-12 and TNFα responses were unaffected [57]. Whether these discrepancies with our present observations are related to cell-specific effects or variations in effects of AhR in different target genes remains to be clarified.

## Conclusion

Understanding the role of AhR and Arnt in the regulation of inflammation may be crucial to elucidate the pro-inflammatory effects of complex chemical mixtures, such as air pollution which may contain a variety of organic chemicals with varying affinity for the AhR [58]. Moreover, individual variation in AhR and Arnt expression levels appear to be considerable [59],[60] and may possibly affect susceptibility towards inflammatory disease. Though it remains to unravel the precise mechanisms by which AhR and its partners modulate inflammation, our present results suggest that depending on the type and combination of stimuli AhR may possess both pro- and anti-inflammatory functions, even within a single cell type. Both constitutive and ligand-activated AhR may elicit a weak to moderate pro-inflammatory signal, but seems to restrict activation of p65 upon encounters with strong activators of the classical NF-κB pathway, such as Poly I:C or TNF-α. The AhR binding partner Arnt displays a separate anti-inflammatory role. While AhR appears to attenuate the onset of p65 activation, including phosphorylation at Ser536, Arnt may possibly block the access of activated p65 to kB-sites in NF-κB-regulated promoters (Figure 10). This would fit with the traditional perception of a differential intracellular localization of AhR and Arnt in cytosol and nucleus of resting cells, respectively [9]. However, Arnt may also be present in the cytosol [49], and our current findings suggest that AhR and Arnt were relatively evenly distributed across the two compartments in unstimulated BEAS-2B cells. Finally, our results suggest that both unligated and ligand-activated AhR also suppress pro-inflammatory responses through pathways other than NF-κB. Thus, AhR and Arnt appear to play complex many-faceted roles in the regulation of pro-inflammation reactions in lung cells.

## Materials and methods

### Reagents

Culture medium, Nutrition Mixture F12 HAM Kaigin’s modification (F12K) was obtained from Sigma–Aldrich, LHC-9 medium was from Invitrogen, while RPMI 1640 and fetal bovine serum (FBS) were from Gibco BRL. Ampicillin and fungizone were from Bristol-Myer Squibb, and penicillin/streptomycin was from Bio Whittaker. B[*a*]P, 1-NP, 1-AP, α-naphthoflavone (ANF), β-naphthoflavone (BNF), dimethyl sulphoxide (DMSO), Poly I:C-potassium salt, antibodies against β-actin, short interfering RNA (siRNA) against AhR (sense: GGACAAACUUUCAGUUCUU, anti-sense: AAGAACUGAAAGUUUGUCC) and RelB (sense: GACAAGAAAUCCACAAACA, anti-sense: UGUUUGUGGAUUUCUUGUC) and non-targeting control siRNA (sense: UAGCGACUAAACACAUCAA, anti-sense: UUGAUGUGUUUAGUCGCUA) were from Sigma-Aldrich. SiRNA against Arnt (ON-TARGET plus Smart Pool L-007207-00-0005) was from Dharmacon RNAi Technologies (Thermo Fischer Scientific). Antibodies against AhR, Arnt, RelB and p52, and siRNA against p52 (sc-29409) with corresponding non-targeting control siRNA (sc-37007) were from Santa Cruz Biotechnology. Antibodies against p65, phospho-p65 (Ser536), IκB, histone H1 and GAPDH, ChIP grade antibody against p65 (D14E12), and SiRNA against p65 (SignalSilence® NF-κB p65 siRNA I #6261) with corresponding non-targeting control siRNA (SignalSilence® Control siRNA #6568) were from Cell Signaling Technology. ChIP grade antibodies against AhR (ChIP grade ab2) and Arnt (ChIP grade ab2770) were from Abcam plc. All other chemicals used were purchased from commercial sources at the highest purity available.

### Cell cultures

BEAS-2B cells, a SV40 hybrid (Ad12SV40) transformed human bronchial epithelial cell line, were from European Collection of Cell Cultures. Cells were maintained in serum-free LHC-9 medium in collagen-coated (PureColTM, Inamed Biomaterials) flasks. The human alveolar type-II-like carcinoma cell line A549 and the human leukemia monocyte cell line THP-1 were obtained from the American Type Culture Collection (Manhasset, VA, USA). A549 cells were cultured in F12K medium, supplemented with ampicillin (100 μg/ml), penicillin/streptomycin (100 μg/ml), fungizone (0.25 μg/ml) and 10% heat-inactivated FBS, while THP-1 cells were cultured in RPMI 1640 medium supplemented with 100 μg/ml gentamicin and 10% heat-inactivated FBS fetal calf serum. Cells were cultured in a humidified atmosphere at 37°C with 5% CO_2_, and passaged twice per week. Prior to exposure, cells were plated in 12-well culture dishes, grown to near confluence and treated as described elsewhere. When used, DMSO concentrations in all samples were below 0.5%.

### Gene expression

Total RNA was isolated using Absolutely RNA Miniprep Kit (Stratagene, La Jolla, CA, USA) and reverse transcribed to cDNA on a PCR System 2400 (PerkinElmer) using a High Capacity cDNA Archive Kit (Applied Biosystems). Real-time PCR was performed using pre-designed TaqMan Gene Expression Assays and TaqMan Universal PCR Master Mix and run on ABI 7500 fast (Applied Biosystems). Gene expression of CXCL8 (Hs00174103_m1), CCL5 (Hs00174575_m1) and CYP1A1 (Hs00153120_m1) were normalized against 18S rRNA (Hs99999901_s1), and expressed as fold change compared to untreated control as calculated by the ΔΔCt method (ΔCt = Ct [Gene of Interest] – Ct [18S]; ΔΔCt = ΔCt [Treated] – ΔCt [Control]; Fold change = 2 ^[−ΔΔCt]^).

### Chemokine release

Chemokine protein levels in cell-supernatants were determined by ELISA assays for CXCL8 (Human IL-8 Cytoset) and CCL5 (Human RANTES Cytoset) from Biosource International as described elsewhere [24]. Absorbance was measured using a plate reader (TECAN Sunrise, Phoenix Research Products) complete with software (Magellan V 1.10).

### Gene silencing by siRNA

BEAS-2B cells were reverse-transfected with the respective siRNAs, using HiPerFect transfection reagent according to the Fast-Forward protocol for adherent cells recommended by Qiagen adapted for 12-well plate format, to give a final siRNA concentration of 10 nM and 2.75 μl of HiPerFect in a total of 1 ml growth medium. The effectiveness of gene silencing was monitored at 48, 72 and 96 h by measuring protein levels by Western blotting.

### NF-κB luciferase assay

Cells seeded at 55x10^3^ cells/cm^2^ were transfected 24 h later with the respective siRNAs using DharmaFECT 1 (Thermo Fischer Scientific) according to protocol. The next day, reporter gene constructs were transiently transfected into cells. For this, the media were changed to opti-MEM (Gibco) containing 2.5 μl/ml of Fugene 6 (Roche), 375 ng of a NF-kB-driven luciferase reporter vector (Stratagene ref: 219058–51) and 40 ng of a renilla reporter vector, used as an internal control, according to the manufacturer’s instructions. 24 h after this second transfection, cells were exposed for 6 or 16 h to DMSO or 1-NP or TNFα. Cell extracts were assayed for luciferase activity with a Dual-Luciferase® Reporter assay system from Promega according to the manufacturer’s instructions.

### Immunoblotting

Total and phosphorylated protein levels were analyzed by Western blotting as described elsewhere [61]. The Compartment Protein Extraction Kit (BioChain Institute, Inc.) was used for or extraction/isolation of nuclei and cytosol according to producer’s recommendations. The blots were developed and quantified using a ChemiDox™ XRS + molecular imager with Image Lab™ software, Bio-Rad Laboratories Inc. (Hercules, CA, USA).

### Immunocytochemistry

Cells were grown in X-well Tissue Culture Chambers (Sarstedt). Effect of treatment were stopped by washing with ice-cold PBS, and cells were fixed by incubation inn methanol for 4 min, before incubation overnight with antibodies against p65, AhR or Arnt (working dilution 1: 200 in PBS with 1% BSA). After washing and incubating for 3 h with secondary antibodies conjugated with Alexa Fluor®488 or 594, the preparations were visualized using a Zeiss AxioObserved.Z1 equiped with an AxioCam ERc 5 s digital camera (Zeiss).

### Chromatin immunoprecipitation (ChIP) assay

ChIP assay was performed according to the protocol specified by the manufacturer (HighCell# ChIP kit, Diagenode Inc.), In brief, treated cells were collected by trypsinisation, and DNA-protein interactions were cross-linked with 1% formaldehyde. Chromatin shearing was performed by sonication at 4°C with a Bioruptor® Plus equipped with a Minichiller (Diagenode Inc.). Lysates were centrifuged, and aliquots (1%) of the supernatants were collected for input control, while the remaining supernatants were incubated overnight (at 4°C) with magnetic beads coated with ChIP grade antibodies against p65, AhR or Arnt. DNA was isolated from the collected precipitates after reversal of the cross-linking by incubation for 15 min at 55°C. Real-time PCR were performed using primer sets for human CXCL8 and CCL5 promoter regions containing NF-κB response elements [62],[63]. The sequences of primers used for ChIP assay were as follows: human CXCL8 sense 5’-AGTGTGATGACTCAGGTTTGCCCT-3’ and anti-sense 5’-AAGCTTGTGTGCTCTGCTGTCTCT-3’; human CCL5 promoter sense 5’-GGGAAGAAGATTGCCTAAAC-3’ and antisense 5’-TGTGGAAATCAAAGGGACAG-3’.

### Statistical analysis

Statistical significance was evaluated by GraphPad Prism software (GraphPad Software Inc., San Diego, CA, USA), using analysis of variance (ANOVA) with Bonferroni post-test.

## Competing interests

The authors declare that they have no competing interests.

## Authors’ contributions

JØ participated in conceiving and designing the study, carried out the majority of experiments, performed the statistical analysis and drafted the manuscript. VL and DG participated in designing the siRNA experiments and drafting of the manuscript. VL also carried out the luciferase assay. TS carried out the studies in A549 cells and participated in drafting the manuscript. RB performed the immunocytochemistry and participated in drafting the revised manuscript. ML, MR, PES and JAH participated in the conceiving and design of the study and helped to draft the manuscript. DLG participated in the design of the study, helped to draft the manuscript, and together with JAH coordinated the collaboration between NIPH and the Inserm institute/University of Rennes. All authors read and approved the final manuscript.

## Additional files

## Supplementary Material

Additional file 1: Figure S1. AhR, Arnt, p65 and RelB differentially regulate basal CXCL8 andCCL5 levels in BEAS-2B cells. Cells were transfected with siRNA against AhR (siAHR), Arnt (siARNT), p65 (siP65), RelB (siRELB) or non-targeting control siRNA (siNT). Basal levels of CXCL8 (A, C and E) and CCL5 (B, D and F) protein levels in the medium (produced over a period of 18 h) were measured by ELISA as described under “Materials and methods”. The results are expressed as mean ± SEM (n ≥6). *Significantly different from cell transfected with non-targeting siRNA. Click here for file

Additional file 2: Figure S2. Poly I:C induce both CXCL8 and CCL5 responses and mediates IκB degradation and p65 phosphorylation in BEAS-2B cells. Cells were exposed different concentrations of Poly I:C for 18 h, and CXCL8 (A) and CCL5 (B) levels in the medium were measured by ELISA as described under “Materials and methods”. Subsequently, cells were exposed to 20 μM 1-NP, 1-AP or 10 μg/ml of Poly I:C for 2 and 4 h (n = 2). Intracellular protein levels of IκB, β-actin, p65 and phosphor-p65 (Ser536) were detected by Western blotting. The figure displays representative blots as well as changes in IκB and phospho-p65 (p-p65) relative to β-actin or total p65, respectively, as quantified by densitometric analysis of the Western blots. The results are expressed as mean ± SEM (n = 2). Click here for file

Additional file 3: Figure S3 AhR suppress CXCL8 and CCL5 responses in Poly I:C-exposed BEAS-2B cells. Cells were transfected with commercially available siRNA (Dharmacon RNAi Technologies, Thermo Fischer Scientific) against AhR (siAHR; ON-TARGET plus Smart Pool L-004990-00) or non-targeting control siRNA (siNT; ON-TARGET Control Pool D-001810-10-05), and exposed to 10 μg/ml Poly I:C. CXCL8 (A) and CCL5 (B) protein levels in the medium were measured by ELISA after 18 h exposure, as described under “Materials and methods”, and expressed as fold increase compared to untreated controls transfected with siNT. The results are expressed as mean ± SEM (n = 2-3). Click here for file

Additional file 4: Figure S4. Sub-cellular localization of p65, AhR and Arnt in 1-NP and Poly I:C exposed BEAS-2B cells. Cells were exposed to 1-NP (20 μM) or Poly I:C (10 μg/ml) for 2.5 h. Sub-cellular localization of p65, AhR and Arnt were detected by immunocytochemistry with specific antibodies, as described under “Materials and methods”. Click here for file

Additional file 5: Figure S5. Characterization of the role of alternative NF-κB signaling in CXCL8 and CCL5 responses in BEAS-2B cells. Cells were transfected with siRNA against p100/p52 (siP52) or non-targeting control siRNA (siNT), and exposed to 20 μM 1-NP, 1-AP or 10 μg/ml Poly I:C for 18 h. CXCL8 (A) and CCL5 (B) levels in the medium were measured by ELISA as described under “Materials and methods”. Efficiency of transfection with siP52 was assessed by Western blotting (C). Subsequently, non-transfected cells were exposed to 20 μM 1-NP, 1-AP or 10 μg/ml of Poly I:C for 2 and 4 h. Intracellular protein levels of p100, p52 and β-actin were detected by Western blotting (D). The figure presents results obtained from 1 experiment. Click here for file

Additional file 6: Figure S6. AhR-activation suppresses Poly I:C-induced chemokine responses in A549 and THP-1 cells. A549 (A and C) and THP-1 (B and D) cells were incubated with 1 μM of the AhR agonist β-naphtoflavone (BNF) for 30 min prior to exposure to 10 μg/ml Poly I:C for 18 h. CXCL8 (A and B) and CCL5 (C and D) levels in the medium were measured by ELISA as described under “Materials and methods”. *Significant increase induced by Poly I:C; †Significant reduction induced by BNF. The results are expressed as mean ± SEM (1 experiment performed in triplicates). Click here for file

Additional file 7: Figure S7. Pre-treatment with B[*a*]P suppress LPS and Poly I:C induced chemokine responses in BEAS-2B cells. Cells were incubated with 25 μM B[*a*]P for 2 h or 24 h prior to exposure to 10 μg/ml LPS or Poly I:C for 18 h. CXCL8 (A) and CCL5 (B) levels in the medium were measured by ELISA as described under “Materials and methods”. The results are expressed as mean ± SEM (n = 2). Click here for file
