# Supplementary figures and images for: AhR and Arnt differentially regulate NF-κB signaling and chemokine responses in human bronchial epithelial cells
Source: Cell Commun Signal. 2014 Jul 24;12:48. doi: 10.1186/s12964-014-0048-8 (PMC4222560; doi:10.1186/s12964-014-0048-8)

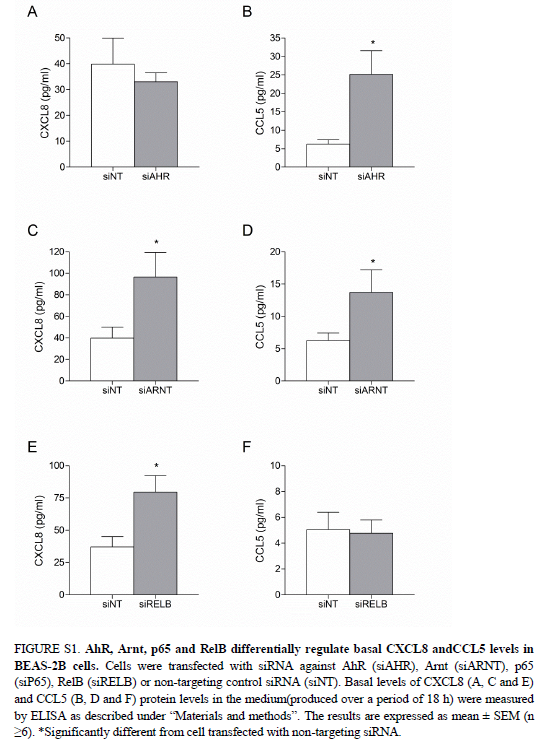

Supplement: Additional file 1: Figure S1. — AhR, Arnt, p65 and RelB differentially regulate basal CXCL8 andCCL5 levels in BEAS-2B cells. Cells were transfected with siRNA against AhR (siAHR), Arnt (siARNT), p65 (siP65), RelB (siRELB) or non-targeting control siRNA (siNT). Basal levels of CXCL8 (A, C and E) and CCL5 (B, D and F) protein levels in the medium (produced over a period of 18 h) were measured by ELISA as described under “Materials and methods”. The results are expressed as mean ± SEM (n ≥6). *Significantly different from cell transfected with non-targeting siRNA. [file s12964-014-0048-8-S1.png]

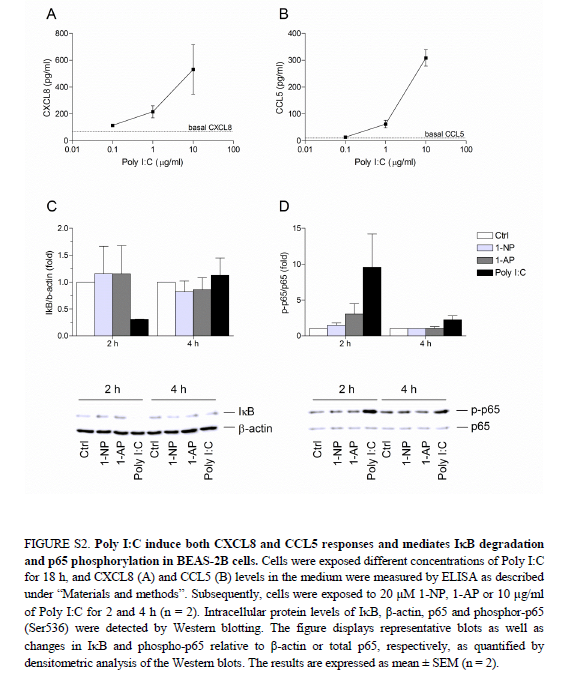

Supplement: Additional file 2: Figure S2. — Poly I:C induce both CXCL8 and CCL5 responses and mediates IκB degradation and p65 phosphorylation in BEAS-2B cells. Cells were exposed different concentrations of Poly I:C for 18 h, and CXCL8 (A) and CCL5 (B) levels in the medium were measured by ELISA as described under “Materials and methods”. Subsequently, cells were exposed to 20 μM 1-NP, 1-AP or 10 μg/ml of Poly I:C for 2 and 4 h (n = 2). Intracellular protein levels of IκB, β-actin, p65 and phosphor-p65 (Ser536) were detected by Western blotting. The figure displays representative blots as well as changes in IκB and phospho-p65 (p-p65) relative to β-actin or total p65, respectively, as quantified by densitometric analysis of the Western blots. The results are expressed as mean ± SEM (n = 2). [file s12964-014-0048-8-S2.png]

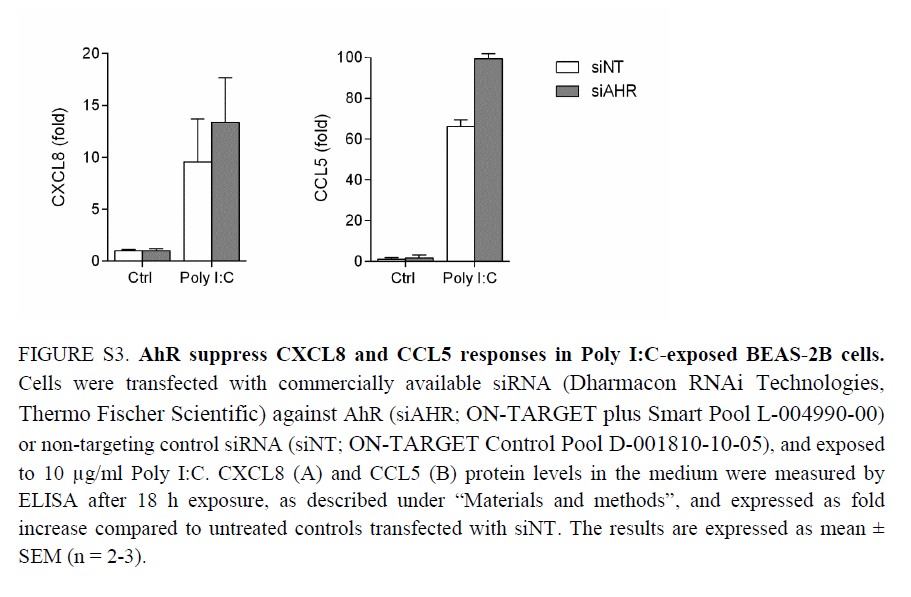

Supplement: Additional file 3: Figure S3 — AhR suppress CXCL8 and CCL5 responses in Poly I:C-exposed BEAS-2B cells. Cells were transfected with commercially available siRNA (Dharmacon RNAi Technologies, Thermo Fischer Scientific) against AhR (siAHR; ON-TARGET plus Smart Pool L-004990-00) or non-targeting control siRNA (siNT; ON-TARGET Control Pool D-001810-10-05), and exposed to 10 μg/ml Poly I:C. CXCL8 (A) and CCL5 (B) protein levels in the medium were measured by ELISA after 18 h exposure, as described under “Materials and methods”, and expressed as fold increase compared to untreated controls transfected with siNT. The results are expressed as mean ± SEM (n = 2-3). [file s12964-014-0048-8-S3.png]

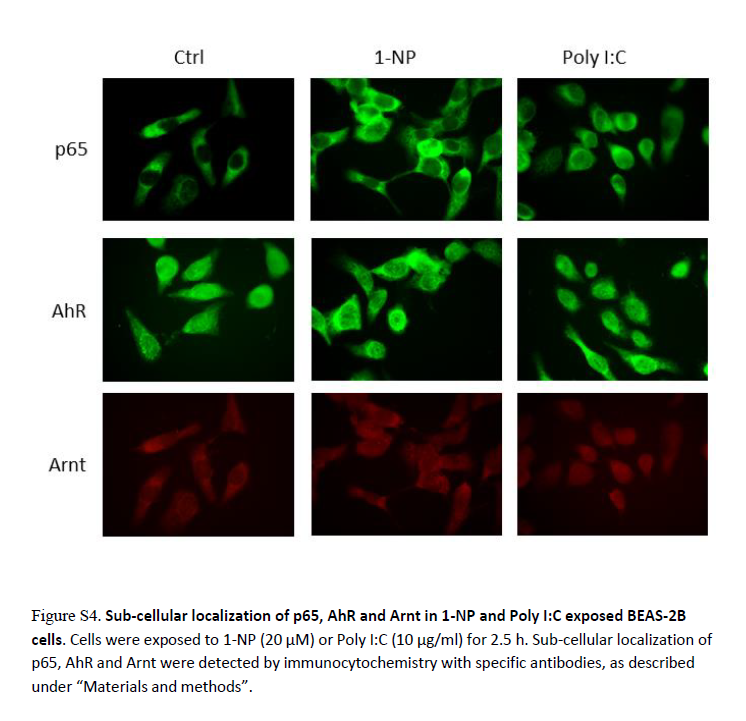

Supplement: Additional file 4: Figure S4. — Sub-cellular localization of p65, AhR and Arnt in 1-NP and Poly I:C exposed BEAS-2B cells. Cells were exposed to 1-NP (20 μM) or Poly I:C (10 μg/ml) for 2.5 h. Sub-cellular localization of p65, AhR and Arnt were detected by immunocytochemistry with specific antibodies, as described under “Materials and methods”. [file s12964-014-0048-8-S4.png]

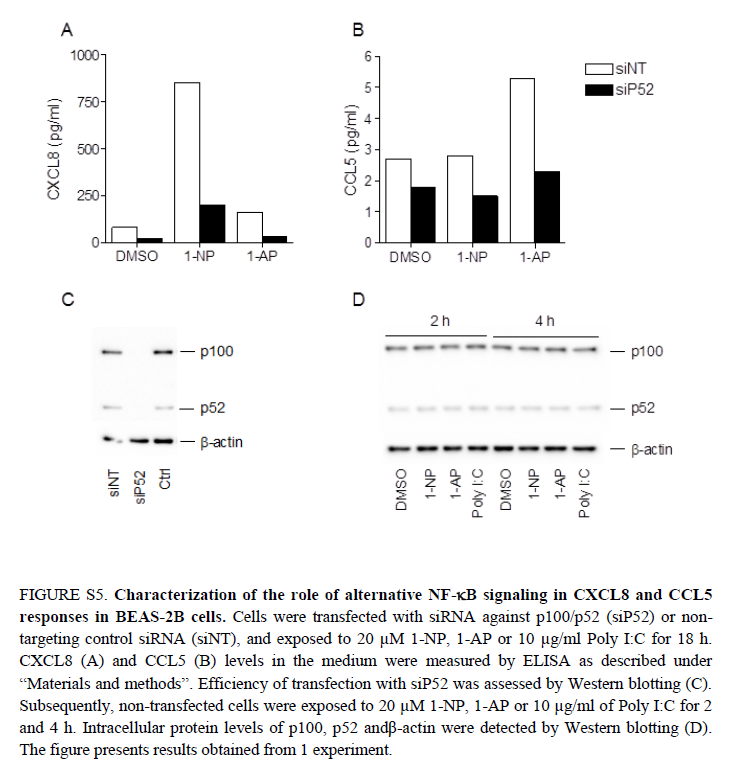

Supplement: Additional file 5: Figure S5. — Characterization of the role of alternative NF-κB signaling in CXCL8 and CCL5 responses in BEAS-2B cells. Cells were transfected with siRNA against p100/p52 (siP52) or non-targeting control siRNA (siNT), and exposed to 20 μM 1-NP, 1-AP or 10 μg/ml Poly I:C for 18 h. CXCL8 (A) and CCL5 (B) levels in the medium were measured by ELISA as described under “Materials and methods”. Efficiency of transfection with siP52 was assessed by Western blotting (C). Subsequently, non-transfected cells were exposed to 20 μM 1-NP, 1-AP or 10 μg/ml of Poly I:C for 2 and 4 h. Intracellular protein levels of p100, p52 and β-actin were detected by Western blotting (D). The figure presents results obtained from 1 experiment. [file s12964-014-0048-8-S5.png]

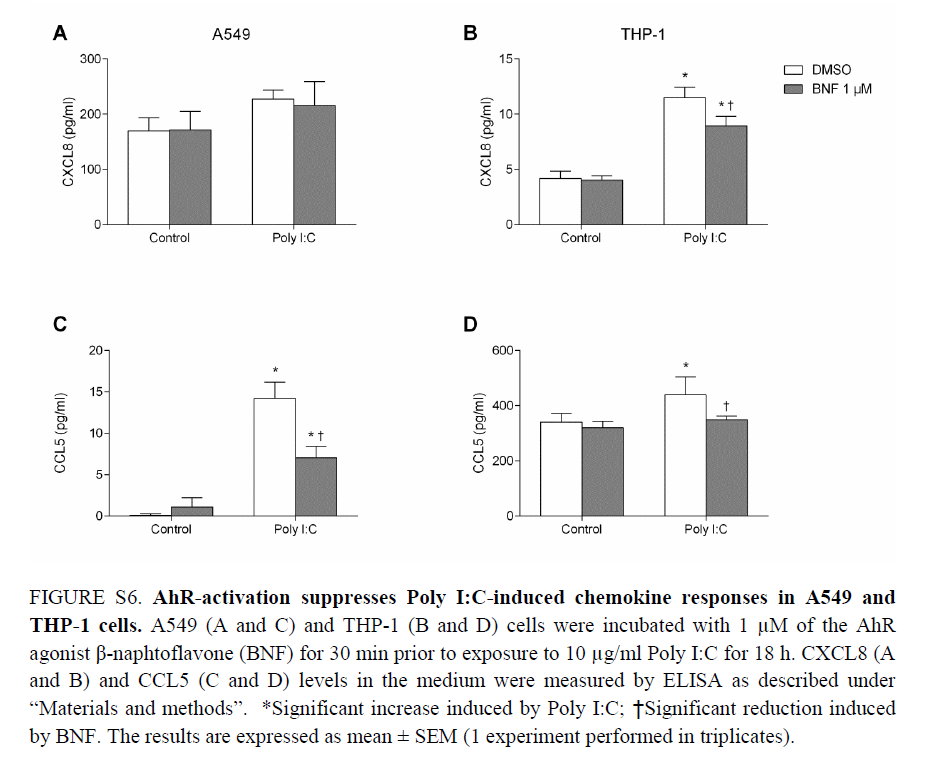

Supplement: Additional file 6: Figure S6. — AhR-activation suppresses Poly I:C-induced chemokine responses in A549 and THP-1 cells. A549 (A and C) and THP-1 (B and D) cells were incubated with 1 μM of the AhR agonist β-naphtoflavone (BNF) for 30 min prior to exposure to 10 μg/ml Poly I:C for 18 h. CXCL8 (A and B) and CCL5 (C and D) levels in the medium were measured by ELISA as described under “Materials and methods”. *Significant increase induced by Poly I:C; †Significant reduction induced by BNF. The results are expressed as mean ± SEM (1 experiment performed in triplicates). [file s12964-014-0048-8-S6.png]

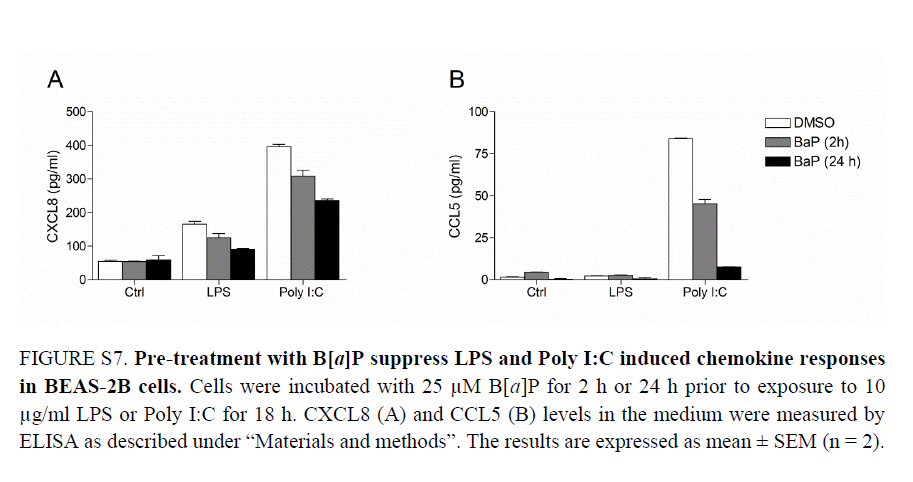

Supplement: Additional file 7: Figure S7. — Pre-treatment with B[a]P suppress LPS and Poly I:C induced chemokine responses in BEAS-2B cells. Cells were incubated with 25 μM B[a]P for 2 h or 24 h prior to exposure to 10 μg/ml LPS or Poly I:C for 18 h. CXCL8 (A) and CCL5 (B) levels in the medium were measured by ELISA as described under “Materials and methods”. The results are expressed as mean ± SEM (n = 2). [file s12964-014-0048-8-S7.png]
